# Application of Multilayer Perceptron with Automatic Relevance Determination on Weed Mapping Using UAV Multispectral Imagery

**DOI:** 10.3390/s17102307

**Published:** 2017-10-11

**Authors:** Afroditi A. Tamouridou, Thomas K. Alexandridis, Xanthoula E. Pantazi, Anastasia L. Lagopodi, Javid Kashefi, Dimitris Kasampalis, Georgios Kontouris, Dimitrios Moshou

**Affiliations:** 1 Agricultural Engineering Laboratory, Faculty of Agriculture, Aristotle University of Thessaloniki, 54124 Thessaloniki, Greece; tamouridoualex@gmail.com (A.A.T.); renepantazi@gmail.com (X.E.P.); 2 Laboratory of Remote Sensing and GIS, Faculty of Agriculture, Aristotle University of Thessaloniki, 54124 Thessaloniki, Greece; thalex@agro.auth.gr (T.K.A.); dkasampa@agro.auth.gr (D.K.); giorgoskontouris@gmail.com (G.K.); 3 Plant Pathology Laboratory, Faculty of Agriculture, Aristotle University of Thessaloniki, 54124 Thessaloniki, Greece; lagopodi@agro.auth.gr; 4 USDA-ARS-European Biological Control Laboratory, 54623 Thessaloniki, Greece; jkashefi@ars-ebcl.org

**Keywords:** remote sensing, precision agriculture, crop monitoring, data fusion

## Abstract

Remote sensing techniques are routinely used in plant species discrimination and of weed mapping. In the presented work, successful *Silybum marianum* detection and mapping using multilayer neural networks is demonstrated. A multispectral camera (green-red-near infrared) attached on a fixed wing unmanned aerial vehicle (UAV) was utilized for the acquisition of high-resolution images (0.1 m resolution). The Multilayer Perceptron with Automatic Relevance Determination (MLP-ARD) was used to identify the *S. marianum* among other vegetation, mostly *Avena sterilis* L. The three spectral bands of Red, Green, Near Infrared (NIR) and the texture layer resulting from local variance were used as input. The *S. marianum* identification rates using MLP-ARD reached an accuracy of 99.54%. Τhe study had an one year duration, meaning that the results are specific, although the accuracy shows the interesting potential of *S. marianum* mapping with MLP-ARD on multispectral UAV imagery.

## 1. Introduction

Weed detection has conventionally been carried out through either ground-based platforms or remotely sensed images acquired from aircrafts, unmanned aerial vehicles (UAV) or satellites [1,2] through examination of remote sensing pixel data unable to provide precise crop detection due to pixel variation and spectral behavior resemblance [3]. The main aim of the current study is to provide a robust solution in utilizing remote sensing data for the purpose of plant species identification to contribute to the larger field of precision agriculture practices.

*Silybum marianum* is a weed accountable for major loss of crop yield and is tough to eradicate. Herbicides are costly and pollute the rural and natural ecosystems. Due to its height and thorny leaves, it may also inhibit the movement of livestock across grasslands. Khan et al. (2011) [4] confirmed the allelopathic effects of cold water extracts of *Silybum marianum* triggering harmful effects on germination percentage, germination stage, germination index and seed vigor index on *Phaseolus vulgaris*, *Vigna radiata*, *Cicer arietinum*, and *Glycine max*. For these reasons, it is significant to map the levels and distribution of the weed's presence so as to define a suitable management practice.

Previous successful applications of UAV in weed mapping include Tamouridou et al. (2017) [5] who evaluated the optimum scale for mapping weed patches; Pena 2013 [6] who described weed mapping in early-season maize fields using object-based analysis of UAV images; Torres-Sanchez (2013) [7] who provided configuration and specifications of an UAV for early site specific weed management.

Lamb and Brown (2001) [8] have observed significant variance among plant species rendering separation between weeds and crop plants problematic. Slaughter et al. (2004) [9] studied the spectral reflectance of field- grown tomato and nightshade leaves, which are difficult to discriminate since they belong to the same taxonomic family (Solanaceae). They proved that a broadband Red-Green-Blue (RGB) color classifier reached an accuracy of 76% for species identification in leave-one-out cross- validation tests. Moreover, accurate species recognition (98% or higher) in leave-one-out cross-validation tests was achieved by narrowband classifiers using near-infrared reflectance.

In order to attain real-time recognition of goal plants and to spread over specific application of various management practices in a more effective and specific way, sensors and machine architectures were utilized [10]. Weed species recognition with sensors [11] is critical for the application of required chemical herbicide and spraying dosage. Discrimination between crops and weeds based on spectral leaf reflectance, has been tried in previous studies. Borregaard et al. (2000) [12] showed that it is possible to successfully discriminate among crop and plants as well as between weed species. Additional approaches targeting at weed recognition have been presented, taking into account the leaf size and shape analysis. A method for automatic machine vision based on weed species grouping via active shape modelling (ASM) has reached a correctness between 65% and 90%. Moreover, it has been revealed that spectral reflectance characteristics are adequate to discriminate between different weed species as in Moshou et al. (2001) [13] who realised a successful discrimination between maize crop and weeds with an accurate classification rate of 96% for maize and 90% for weeds. Regarding the discrimination of sugar-beet from weeds, the precision rate was 98% and 97% respectively. Nonetheless, these results pointed solely at crop- weed perception, not weed classes recognition.

Apart from ASM approaches, various classification algorithms are applied in remote sensing mapping applications. In several studies the effectiveness of Artificial Neural Networks (ANN) when applied in remote sensing classification is indicated [14]. The MLP classifiers are networks where the nodes receive inputs coming from previous layers. As a result, the information flows in a single direction to the output layer [14]. In the intermediate layer(s) the number of nodes is related to the complexity and the performance of a neural network model to express the upcoming relationships and structures built-in a training data set which describe the generalization capability of the network [15]. The number of nodes in hidden layers is relevant to achieve classification of more complex, and low quality satellite images.

The current work proposes a data fusion approach directed on weed recognition. By using MLP-ARD *S. marianum* was identified among other vegetation. The data fusion approach consisted of combining the spectral and textural features derived from a UAV multispectral camera.

## 2. Materials and Methods

In the present paper, the proposed MLP-ARD classifier was evaluated for performing fusion of features from UAV images for the accurate recognition of *S. marianum*. The imaging of *S. marianum* was achieved by using a fixed wing UAV and a multispectral camera. The fusion structures that were used were constructed from the mixture of spectral and textural information. The MLP-ARD classifier was used to categorise the data into *S. marianum* and other plants.

To assess the capability of weed recognition, spectral signatures were gathered during a field operation, both from *S. marianum* plants and from other vegetation on 29 May 2015 using a UniSpec-DC portable spectroradiometer (PP Systems, Inc., Amesbury, MA, USA), with a resolution of 10 nm in the range of 310 nm and 1100 nm. From the various plant species, four spectral signatures were taken in the field from the upper surface area of the leaves.

### 2.1. Study Area

**Figure 1:**
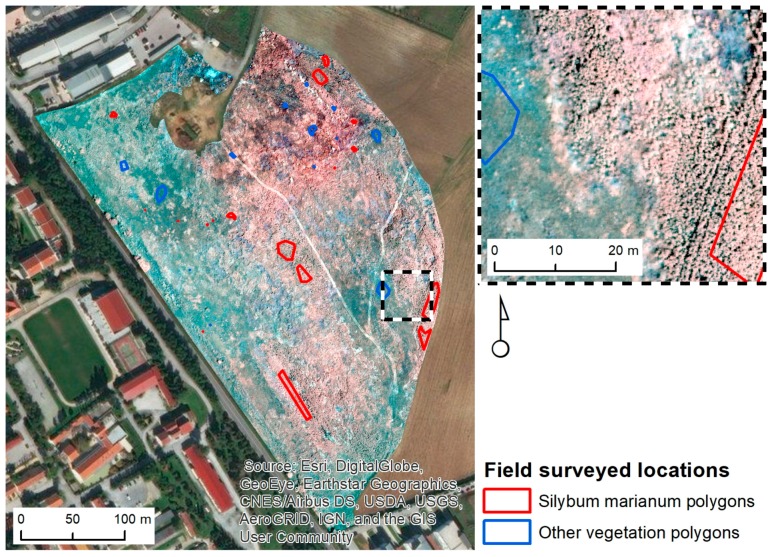
Orientation map and field surveyed locations in the study area.

This study was performed at a 10.1 ha field located in the area of Thessaloniki, Greece (40°34′14.3″ N 22°59′42.6″ E) (Figure 1). The ground topography is about level and the elevation is 75 m. This field belongs to the American Farming School of Thessaloniki and for the past decade it has never been cultivated. In this left aside field, graminaceous weeds are growing, which comprise the field’s main vegetation with huge patches of *S. marianum*. Weeds include: *Avena sterilis* and *Conium maculatum* L.

### 2.2. Preparation of Datasets

Since the study focuses on the efficient combination of spectral and textural information, the reference data gathering process took place in the study areas and is described below. An eBee fixed wing UAV (http://www.geosense.gr/en/ebee/) with a Canon S110 NIR camera (12 Mpixels) acquired the remote sensing images on 19 May 2015. The spectral bands included are green (560 ± 25 nm), red (625 ± 45 nm) and near-infrared (850 ± 50 nm), the original resolution was 0.1 m, and was rescaled to 0.5 m, as demonstrated by Tamouridou et al. (2017) [5]. Beyond the analysis of the spectral information, a texture layer was created based on the NIR layer using the local variance algorithm (moving window size 7 × 7 pixels) depicting the structural patterns of vegetation.

Data was required in order to identify and pinpoint different vegetation categories represented in the UAV image. During a field operation using a handheld Trimble GPS, the location of the largest *S. marianum* patches (which are visible and easily identified) and other predominant vegetation categories were identified. Their locations were logged in the GPS and were used during the construction of the training sets that were fed to the MLP-ARD for image classification. The GPS model that was used in the process, is the GPS Trimble GeoXH 2008 (Sunnyvale, CA, USA). It features EGNOS error correction, which is a combination of field computer Trimble GPS with Microsoft Windows Mobile version 6 software. GeoXH utilizes EVEREST and H-Star™ technology in order to achieve an >30 cm accuracy. Each of the pixels of the UAV image contains specific spectral values corresponding to the afore mentioned spectral regions (Green, Red, NIR). The MLP-ARD algorithm is looking for similarities in those spectral values in order to successfully classify each pixel. The location of the sampling of the two vegetation categories is depicted in the polygons of Figure 1. The polygons were carefully positioned in order to represent areas of the whole region. The pixels from those polygons were used to construct the datasets. The first group of polygons, representing *S. marianum*, consisted of 17 polygons, with a total area of 0.12 ha. The second group, representing the other vegetation types, consisted of 13 polygons with a total area of 0.04 ha.

The afore mentioned polygons were used for the construction of data sets. The constructed datasets comprised of 4745 pixels of the *S. marianum* category and of 1434 pixels of the category of other types of vegetation respectively (Figure 1). The two calibration datasets comprised of 2868 pixels for acquiring equal datasets of *S. marianum* and other vegetation categories. From the calibration set a training dataset was formed containing a random selection of 70% (2008 pixels) for model calibration and setting the rest 30% (860 pixels) for validation.

**Figure 2:**
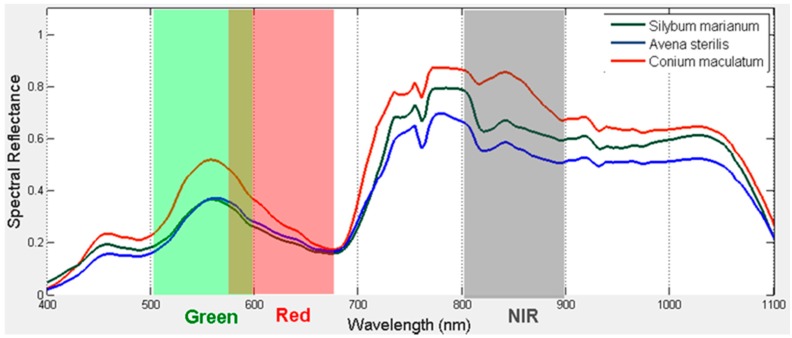
Vegetation species demonstrated similar spectral reflectance with *Silybum marianum*.

In Figure 2, the spectral signatures of three vegetation species (*Avena sterilis*, *Conium maculatum*), are depicted. *A. sterilis* shows analogous spectral reflectance features with *S. marianum* rendering the separation a challenge. The three species, though, were easier to discriminate between in the NIR spectrum. This suggests that the NIR region, and perhaps other features (e.g., texture) can be utilized to make the class separation possible.

### 2.3. Methods of Data Fusion

The intention of this data processing is to develop a model between spectral and textural information of each MS camera pixel and the label of it as belonging to a *S. marianum* or other plant. MLP is a neural network cosisting of multiple layers of neurons. ARD automatically finds relevant neurons by modulating their connections strength so as to maximise relevant neurons in terms of evidence presented from the data, while a weighted evidence of multifeature sets amounts to fusion since adaptive weights are determined to combine effectively input features.

The main goal is to determine a mapping between spectral and textural input data points of each pixel to the plant identity. IT learns without external supervision. The obstacle is overfitting which is avoided by the bayesian regularization applied during learning which restricts ovefitting.

A feed-forward multilayer perceptron neural network (MLP) with one hidden layer and output unit [16] was used by using a given training set:

(1) $$D={\{x_{i},t_{i}\}}_{i=1}^{N}$$

where *x_i_* represents the observation data $x_{i}=\left(x_{i}^{\left(1\right)},\cdots ,x_{i}^{\left(n\right)}\right)\in \;R^{n}$ while *t_i_* is the target category labels $t_{i}\in \;\{0,1\}$. As result, the MLP classification system forms the following non-linear mapping:

(2) $$y(x)=f_{2}\left(\upsilon \;f_{1}\left(u\;x\right)\right)$$

where y(x)∈ R, and υ and u are the respective weight vectors of the hidden and output layer, that establish the weight vector ω. These are estimated during the training process, while hyperbolic transfer functions $f_{1}$ and $f_{2}$ are used for complex non-linear mapping and inserted to the hidden layer. Then, a logistic transfer function is employed in the output layer which facilitates the output of the MLP which can be defined as *y*(*x*) for estimating the form p(t=1|x) in which ω is randomly adapted and incrementally adjusted to minimize a cross-entropy objective function G [16]:

(3) $$G=-\sum_{i=1}^{N}\{t_{i}\ln\left(y_{i}\right)+\left(1-t_{i}\right)\ln\left(1-y_{i}\right)\}$$

For avoiding over-fitting of the objective function regularization weight is realized by the following function:

(4) $$F\left(\omega \right)=G+\sum_{k}\alpha_{k}Ew_{\left(k\right)}$$

in which $Ew_{\left(k\right)}=\frac{1}{2}\sum_{j}\omega_{j}^{2}$, weights $\omega_{j}\in R$ and j represents an index estimating all weights of weight class W(*k*).

Automatic relevance determination (ARD) attains weight regularization by considering *k* = 1, …, *n* + 3 weight classes inside weight vector ω, each associated with a weight decay hyperparameter (*α_k_*). Each hyperparameter (*α_k_*) is related with each predictor variable *x*(*k*) (*k* = 1, …, *n*) and more explicit, with the weights connected to the hidden neurons. Three extra weight decay hyperparameters including weight classes, are demonstrated: The first, associated with the synaptic connection bias, the second is related to the connections from hidden neurons to the output neuron while the third one is associated with the connection that is linking the hidden layer bias neuron and the output neuron [16].

The above described MLP-ARD was employed in dealing with to the UAV image pixels for identifying *S. marianum* weeds and other vegetation according to the calibration dataset. Accuracy assessment was accomplished by assessing the confusion tables on the validation dataset of 860 pixels (441 were from *S. marianum* and 419 from other vegetation).

## 3. Results

The results from the processing of the pixel data with MLP ARD for *S. marianum* identification vs. other vegetation are accessible in Table 1. It is obvious that the MLP-ARD classifier has managed to classify correctly >99% of the pixels of both categories, and only few pixels (<1%) were misclassified.

**Figure 3:**
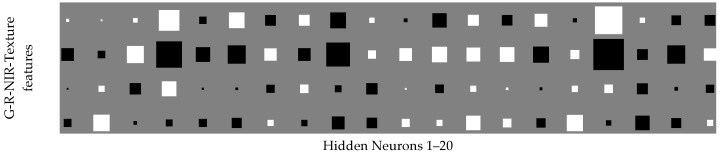
Hinton diagram of the trained MLP-ARD. The vertical axis corresponds to feature while the horizontal shows hidden neurons.

For the visualisation of the MLP-ARD function, the Hinton diagram weight component maps were formed (Figure 3). The Hinton diagram depicts the values of the weights as a combination of spectral and textural features and hidden neurons. Positive weight is depicted in white colour while negative weight is depicted in black. It can be assumed that the weights of less relevant features have been restricted by ARD, while the weights of more active features have been amplified with the condition that the relative magnitudes of the weights are illustrated by the size of the squares. Thus, the status of individual features can be inferred through the Hinton diagrams.

Figure 3 illustrates how the ARD capability has influenced the weight distribution. It shows larger weights that correspond to NIR and texture features. Moreover, the weights are for most hidden neurons for the NIR and texture of opposite sign which means that the overlap of the two features is minimal and that their activity is synergistic. This result in terms of classification shows strong synergy of the texture and NIR components which means that the classifier performs fusion of the components.

**Figure 4:**
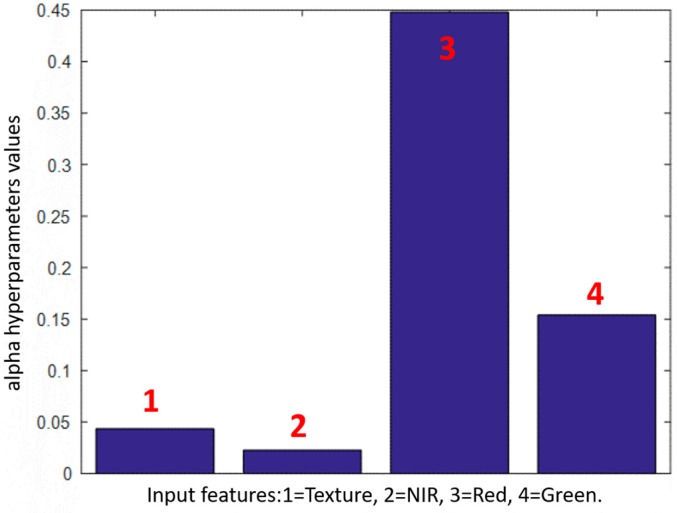
The alpha hyperparameters are shown for the four input features (1 = Texture, 2 = NIR, 3 = Red, 4 = Green).

A quantification of the influence of individual features can be assessed by observation of the L2 norms of the weights and the associated values of the hyperparameters (a) that are calculated from Equation (4). At the final period of the training large norms are associated to smaller hyperparameter values which in turn are linked to larger variances for the weights. In Figure 4 one can assess the values of the hyperparameters for each weight group that link a specific input feature to the units of the hidden layer.

The inverse behaviour of hyperparameters with respect to weights in the trained network is evident. This means that minimal hyperparameters (a) correspond to large weight values. To the contrary, large hyperparameters (a) are associated to weights that are inert so they are not frequently used during training to construct the mapping. In this way, ARD process achieves a soft selection of weights that are more closely related to the classification result. However, the weight groups that link the input layer to the hidden layer following this soft selection can visually depict the influence of each feature has on the classification result. Features that have low importance will remain with large hyperparameters (a) and will have small weights which is a result that is related to their minimal contribution. The opposite occurs when the hyperparameters (a) converge to small values near to zero, indicating large weight values connected to important features.

**Figure 5:**
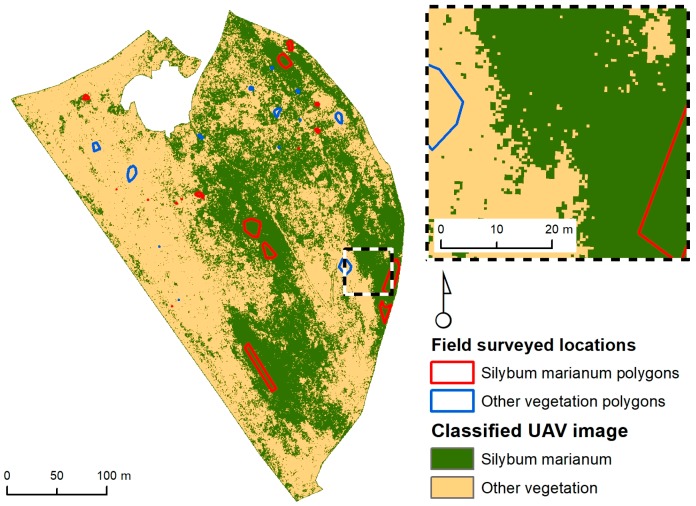
*S. marianum* weed mapping based on MLP-ARD prediction. Green is *S. marianum* and yellow is other vegetation.

The calibration dataset was used to obtain the model. In the case of the whole field the trained model was deployed operationally by using feature vectors from the whole field. The deployment was performed by using new test feature vectors as input to the trained fixed model and obtaining the response of the MLP in the form of binary vectors [1 0] for *S. marianum* and [0 1] for other vegetation. Then, the output vectors were color coded into green and yellow pixels forming the final classification map covering the whole field. The trained MLP-ARD model was fed with feature vectors from the whole field with 782,838 feature vectors formed from UAV images from the whole field and comprising four components (green, red, NIR and texture). The map demonstrates the location of the patches of *S. marianum* and other vegetation in the field so the type of vegetation for each location was not obtained. Each input vector produced a classification corresponding to 1 for *Silybum* and 2 for other vegetation. In Figure 5 the classified UAV image of the study area is shown. The map shown in Figure 5 was produced by the MLP-ARD. Afterwards, in order to validate its success, the aforementioned map was compared to the points of the validation dataset which was sampled to be representative of the whole area. The map's accuracy was found to be 99.55%. Large patches of *S. marianum* is evident on the central and eastern parts of the study area, which are interleaved by large patches of other vegetation types on the south east. Only small sporadic patches of *S. marianum* appear in the western part of the study area.

## 4. Discussion

The regularisation procedure has the potential to constrain the model complexity. For example, a cost function could accommodate an additional function by adding a weight constraining term. Individual constraining terms associated to the weights offer an advantage according to the evidence framework of MacKay [17].

By employing the evidence framework there is no actual need to foresee a validation set. In the current work different weight groups were equipped with individual constraining parameters. Such a regularisation process was employed by implementing the ARD approach through which a soft feature selection was achieved. The term “soft” selection is founded on the idea of maintaining the extra features in the network whereas constraining their influence to minimal values.

The field in question has not been cultivated in the past decade, so the present vegetation consists of endemic weed species growing in random patterns. The vegetation nearby the *S. marianum* patches consists mainly of graminaceous weeds that generate a flat surface with low texture (as observed in Figure 1). On the other hand, the *S. marianum* patches consist of large discrete plants with high texture. This effect explains the positive relation of texture to *S. marianum* appearance.

During the specific season when the field data was obtained, the bulk of the area’s vegetation consisting of *A. sterilis* was already drying up compared to the *S. marianum* weeds that were still vigorous. Therefore, the *S. marianum* patches were the only areas in the field where high NIR reflection was observed. This effect substantiates the positive relation of NIR to *S. marianum* presence. These effects and observations are relevant to the general conditions of the present study (e.g., season, other vegetation) and are not necessarily representative of every situation.

By assimilating all the above it can be decided that both the texture and NIR features have a synergistic fusion effect on the classification accuracy while on the other hand MLP-ARD exploits this synergy by fusing the most active synaptic contributions to accurately classify *S. marianum* presence vs. other vegetation. As shown in Figure 3, the synergistic effect of the two features (NIR and texture) lays on the complementary of their weights, meaning that the classifier performs fusion of the components, thus affirming the main aim of the study which was discrimination of vegetation species by sensor fusion.

## 5. Conclusions

In the current paper it was established that it is possible to map *S. marianum* by using the MLP-ARD classifier. A NIR multispectral camera on a UAV was employed for the acquisition of high-resolution images. As functional input to the classifier, the three spectral bands (green-red-NIR) and the texture feature based on the local variance of the NIR layer were used. The classification rates obtained using MLP-ARD network reached high overall accuracy and reliability by achieving a 99.55% correct *S. marianum* detection. The results indicate that the on-line classification of *S. marianum* with MLP-ARD can be used operationally for performing UAV based weed mapping for various applications, including coverage assessment, eradication programs and assessment of treatment effectiveness by using change detection.

## Figures and Tables

**Table 1:** Confusion matrix of the MLP-ARD for *S. marianum* and other plants. Percentages are estimated on actual observation sums.

Categories	Network Prediction	
	*S. marianum* (%) 441 pixels	Other plants (%) 419 pixels
*S. marianum*	99.55	0.45
Other plants	0.48	99.52
